# Dissecting a hypoxia-related angiogenic gene signature for predicting prognosis and immune status in hepatocellular carcinoma

**DOI:** 10.3389/fonc.2022.978050

**Published:** 2022-08-30

**Authors:** Guixiong Zhang, Yitai Xiao, Xiaokai Zhang, Wenzhe Fan, Yue Zhao, Yanqin Wu, Hongyu Wang, Jiaping Li

**Affiliations:** ^1^ Department of Interventional Oncology, The First Affiliated Hospital, Sun Yat-Sen University, Guangzhou, China; ^2^ Guangdong Provincial Key Laboratory of Biomedical Imaging and Guangdong Provincial Engineering Research Center of Molecular Imaging, The Fifth Affiliated Hospital, Sun Yat-sen University, Zhuhai, China

**Keywords:** hepatocellular carcinoma, hypoxia, angiogenesis, prognosis, immune status

## Abstract

**Background:**

Hypoxia and angiogenesis, as prominent characteristics of malignant tumors, are implicated in the progression of hepatocellular carcinoma (HCC). However, the role of hypoxia in the angiogenesis of liver cancer is unclear. Therefore, we explored the regulatory mechanisms of hypoxia-related angiogenic genes (HRAGs) and the relationship between these genes and the prognosis of HCC.

**Methods:**

The transcriptomic and clinical data of HCC samples were downloaded from public datasets, followed by identification of hypoxia- and angiogenesis-related genes in the database. A gene signature model was constructed based on univariate and multivariate Cox regression analyses, and validated in independent cohorts. Kaplan-Meier survival and time-dependent receiver operating characteristic (ROC) curves were generated to evaluate the model’s predictive capability. Gene set enrichment analysis (GSEA) was performed to explore signaling pathways regulated by the gene signature. Furthermore, the relationships among gene signature, immune status, and response to anti-angiogenesis agents and immune checkpoint blockade (ICB) were analyzed.

**Results:**

The prognostic model was based on three HRAGs (ANGPT2, SERPINE1 and SPP1). The model accurately predicted that low-risk patients would have longer overall survival than high-risk patients, consistent with findings in other cohorts. GSEA indicated that high-risk group membership was significantly associated with hypoxia, angiogenesis, the epithelial-mesenchymal transition, and activity in immune-related pathways. The high-risk group also had more immunosuppressive cells and higher expression of immune checkpoints such as PD-1 and PD-L1. Conversely, the low-risk group had a better response to anti-angiogenesis and ICB therapy.

**Conclusions:**

The gene signature based on HRAGs was predictive of prognosis and provided an immunological perspective that will facilitate the development of personalized therapies.

## Introduction

Globally, liver cancer was the sixth-most common cancer (accounting for 4.5% of all new tumors) and the third leading cause of cancer-related death (accounting for over 8.3% of all such deaths) in 2020 (1). Among the primary types of liver cancer, hepatocellular carcinoma (HCC) accounts for approximately 75–85% of all cases, and the 5-year survival rate of HCC in China is only 14.1% (1, 2). Ablative therapies, transarterial chemoembolization (TACE), and surgery are routine treatments for HCC (3). Because of the complex etiology and high heterogeneity of HCC, its treatments and prognosis are unsatisfactory, and prognosis prediction is challenging (4–6). To improve prognosis, identifying biomarkers for treatment and prognostic prediction of HCC is critical.

Due to tumor neovascularization and high metabolism, hypoxia is present in approximately half of solid tumors, including HCC (7–10). Hypoxia promotes tumor cell proliferation and tumor progression by initiating multiple adaptive behaviors, such as angiogenesis, proliferation, and invasion. Hypoxia reconstructs the tumor immune microenvironment (TIM) by promoting the recruitment of innate immune cells, and interfering with the differentiation and function of adaptive immune cells (11–13). As a solid tumor rich in blood vessels, tumor-driven hypoxia increases the expression of proangiogenic factors leading to abnormal vascular proliferation of HCC, which contributes to tumor growth, invasion, metastasis, and immunosuppression. Tumor susceptibility to angiogenesis has been a hot spot of research recently, and targeted drugs are in clinical use (14, 15). During cancer progression, several proangiogenic cytokines—such as vascular endothelial growth factor A (VEGFA), fibroblast growth factor (FGF) and hypoxia inducible factor-1 (HIF-1)—which contribute to neovasculature sprouting and formation in the tumor microenvironment (TME), are implicated in immune TME remodeling and direct or indirect immune cell regulation (16–18). Nevertheless, the role of hypoxia in liver cancer angiogenesis is unclear. Closely related to angiogenesis, hypoxia regulates a series of genes involved in tumor angiogenesis, leading to the epithelial-mesenchymal transition (EMT) and immune escape, rendering tumor cells more tolerant to the hypoxic microenvironment, and enhancing their proliferation, metastasis, and invasion. Therefore, we analyzed the effect of angiogenesis-related genes under hypoxic conditions on the survival and immune microenvironment of HCC.

We first downloaded mRNA expression profiles and the corresponding clinical data of patients with HCC from public databases. Second, we constructed a prognostic multigene signature based on hypoxia-related angiogenic genes (HRAGs) in The Cancer Genome Atlas (TCGA) cohort, and validated it in an independent cohort. Third, we performed functional enrichment analysis and immune infiltration, as well as anti-angiogenesis and immune checkpoint blockade (ICB) therapy response predictions, to provide guidance for precise and effective HCC treatment.

## Materials and methods

### Patients and datasets

The mRNA expression profiles and corresponding clinical information of HCC patients were obtained from The Cancer Genome Atlas-Liver Hepatocellular Carcinoma dataset (TCGA-LIHC, https://portal.gdc.cancer.gov/, 370 HCC and 50 normal tissue samples), International Cancer Genome Consortium (ICGC, https://dcc.icgc.org/projects/LIRI-JP, 229 HCC tissue samples), and Gene Expression Omnibus (GEO, https://www.ncbi.nlm.nih.gov/geo/query/acc.cgi?acc=GSE14520, 220 HCC tissue samples). The inclusion criteria for the follow-up analysis were HCC confirmed by pathology, available RNA expression data, and complete clinical data (> 30 days of follow-up). In total, 337 hypoxia-related genes (HRGs) and 201 angiogenesis-related genes (ARGs) were acquired by gene set enrichment analysis (GSEA) (hallmark-hypoxia or hallmark-angiogenesis), as well as from the GeneCards database (using the terms *hypoxia* and *angiogenesis*; relevance scores > 4) and from previous reports.

### Development and validation of a prognostic gene signature

The R package limma was used to identify differentially expressed genes (DEGs) between tumor tissues and adjacent nontumorous tissues, according to the criteria of | log 2 (fold-change) | > 1 and false discovery rate (FDR)< 0.05. Next, hypoxia-related angiogenic DEGs (HRAGs) were identified. The Gene Ontology (GO) and Kyoto Encyclopedia of Genes and Genomes (KEGG) functional annotations of these HRAGs were analyzed and visualized using the R package clusterProfiler.

Univariate Cox regression analysis was performed using the R package survival to determine the prognostic value of the DEGs for OS; *P*< 0.05 was considered statistically significant. An interaction network for the prognostic DEGs was generated using the STRING database (https://www.string-db.org/) and entered into a stepwise multivariate Cox regression analysis to identify covariates with independent prognostic value for OS. The risk score was based on the expression of predictive genes and the multivariate Cox regression risk model coefficients, and was calculated as follows:


$$\text{Risk score }=\;\sum_{i=1}^{n}\left(GeneExpressioni\;\times \;Coefi\right)$$

Based on the median risk score or optimal cut-off value, HCC patients were divided into high- and low-risk groups. A Kaplan–Meier (K-M) survival analysis was performed to compare the high- and low-risk groups according to predictive signatures. The utility of prognostic prediction models was evaluated by calculating the areas under the curve (AUC) values of the receiver operator characteristic (ROC) curve using the R package survivalROC. Principal component analysis (PCA) and t-distributed stochastic neighbor embedding (t-SNE) were used to examine the clustering of the signature genes with the prcomp function of the R package stats and Rtsne. The ICGC and GSE14520 cohorts were analyzed to verify the results.

### Prognostic value of the gene signature and construction of a predictive nomogram

Univariate and multivariate Cox regression analyses were performed to identify factors independently associated with OS in the TCGA cohort. To predict the survival of HCC patients, a nomogram was established based on the risk score and other clinical parameters. Calibration curve and time-dependent ROC analyses were performed to assess the accuracy of the nomogram. The nomogram and calibration curves were plotted using the R package rms.

### Molecular characteristics and biological function analysis

GSEA of the MSigDB Collection (h.all.v7.4.symbols.gmt) was performed using GSEA software (version 4.1.0) to detect the set of genes expressed difference between the high- and low-risk groups. For each analysis, 1,000 gene set permutations were performed.

### Estimation of tumor immune microenvironment

xCell (https://xcell.ucsf.edu/), which is based on a deconvolution algorithm, was used to infer immune cell infiltration from RNA-sequencing data. The infiltration scores of 17 immune cells and activities of 13 immune-related pathways were evaluated by single sample gene set enrichment analysis (ssGSEA) using the R package gsva. The normalized gene expression data of the TCGA and ICGC cohorts were uploaded into Sangerbox tools (http://www.sangerbox.com/tool) for bioinformatics analysis. Next, infiltration of cancer-associated fibroblasts (CAFs) was estimated using the MCPcounter and EPIC algorithms.

### Anti-angiogenesis and ICB therapy response prediction and validation

Sensitivity and resistance to anti-angiogenesis drugs were evaluated using the R package pRRophetic. ImmuCellAI was used to predict the response to Immune checkpoint blockade (ICB) therapy (http://bioinfo.life.hust.edu.cn/ImmuCellAI#!/) (19). Immunohistochemical staining was performed as described previously. Briefly, HCC sample slides were deparaffinized and dehydrated by serial immersion in xylene, ethanol, and distilled water. Antigen retrieval was performed using citrate buffer (10 mM, pH 9.0) in a microwave on medium power. After blocking with goat serum at room temperature for 1 h, the tissues were sequentially incubated with an anti-secreted phosphoprotein-1 (SPP1) antibody (1:100, 22952-1-AP; Proteintech, Rosemont, IL, USA) at 4°C overnight and a secondary antibody at room temperature for 1 hour. A slide scanner (3DHistech Ltd., Budapest, Hungary) was used to capture images. Staining intensity, expressed as the H-score (range: 0–300) was automatically quantified using Pannoramic Viewer software (3DHistech Ltd.). Approval was obtained from the Institutional Review Boards of the Research Institute and Hospital National Cancer Center and The First Affiliated Hospital, Sun Yat-Sen University.

### Statistical analysis

Data management and statistical analysis were conducted using R (version 4.1.0; R Development Core Team, Vienna, Austria) and GraphPad Prism software (version 8.3.0; GraphPad Software Inc., San Diego, CA, USA). Student’s *t*-test or the Wilcoxon rank-sum test was used to compare gene expression between two groups. The chi-squared test was used to compare differences in proportions. Survival curves were plotted using the K-M method and compared by log-rank test. A value of *P*< 0.05 was taken to indicate statistical significance.

## Results

### Datasets

**Figure 1:**
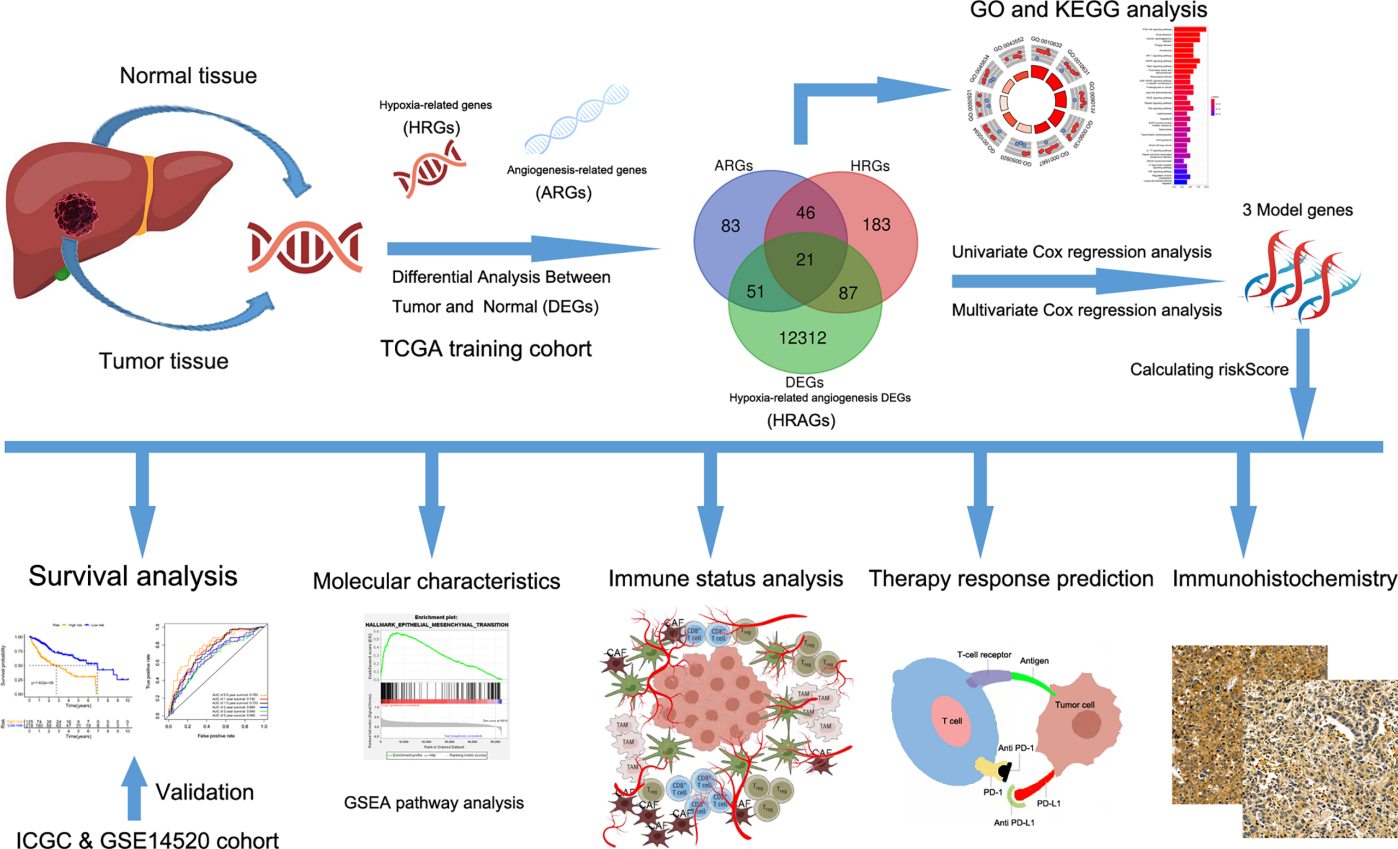
Flow chart of data collection and analysis.

The study flow chart is shown in **Figure 1**. A total of 343 HCC patients from TCGA cohort, 229 from the ICGC cohort, and 220 from the GSE14520 cohort were included in this study. The clinical characteristics of the patients are listed in **Supplementary Table S1**.

### Identification of prognostic hypoxia-related angiogenic genes in the TCGA dataset

**Figure 2:**
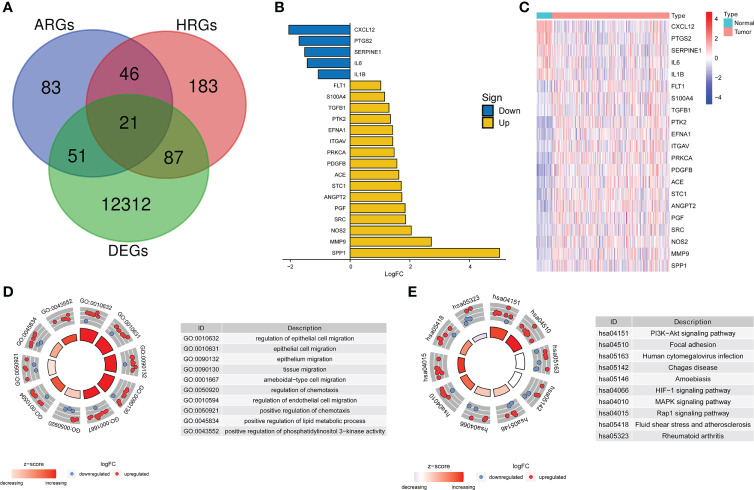
Screening and enrichment analysis of hypoxia-related angiogenesis genes. **(A)** Venn diagram of 21 differentially expressed HRAGs in the TCGA cohort. **(B)** Deviation plot and **(C)** heatmap of 21 differentially expressed HRAGs in HCC and noncancerous tissues. **(D)** GO **(E)** and KEGG analyses revealed the most significantly enriched biological functions and pathways of the overlapping differentially expressed HRAGs.

**Figure 3:**
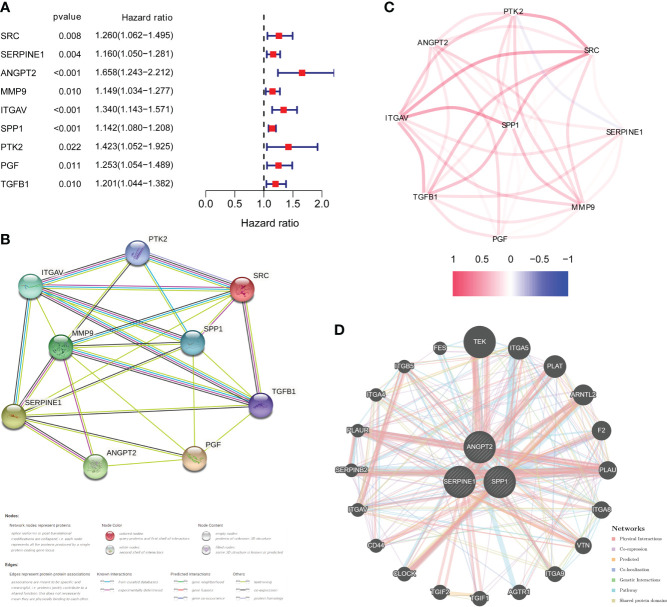
Identification of candidate genes related to the prognosis of HCC. **(A)** Forest plots of univariate Cox regression analysis of gene expression and OS in the TCGA cohort. **(B)** Protein–protein interaction (PPI) network and **(C)** correlated regulation network of prognostic HRAGs. **(D)** Correlation analysis of three model HRAGs from GeneMANIA.

DEGs analysis of the 374 HCC samples and 50 normal liver samples from TCGA revealed 21 differentially expressed HRAGs (**Figures 2A–C**). GO and KEGG enrichment and functional analysis showed that these genes were enriched in epithelial cell migration, chemotaxis, PI3K-Akt signaling pathway, focal adhesion, MAPK signaling pathway, and HIF-1 signaling pathway (**Figures 2D, E**). The HRAGs were related to cancer proliferation and invasion. Univariate Cox regression analysis showed that nine prognostic HRAGs significantly correlated with OS were risk factors for a poor prognosis of HCC (**Figure 3A**). PPI and gene correlation networks suggested connections among the prognostic genes (**Figures 3B, C**).

### Construction and validation of the prognostic model

Three of the nine prognostic HRAGs were used to construct a prognostic predictive model. The corresponding coefficients and gene expression levels were used to calculate the risk score as follows: (0.389932838 × expression level of ANGPT2 + 0.108256217 × expression level of SERPINE1 + 0.121709881 × expression level of SPP1). Patients in the TCGA, ICGC, and GSE14520 datasets were divided into high- and low-risk groups based on the median risk score or optimal cut-off value. Interestingly, K-M survival curves based on the three genes showed that the predicted OS of the low-expression group was significantly longer than that of the high-expression group (**Supplementary Figure S1**). A PPI network was generated based on coexpression information for the three candidate genes from the GeneMANIA database (http://genemania.org/) (**Figure 3D**). The expression of prognostic genes and risk scores were significantly higher for tumor stage III/IV compared with stage I/II patients (**Supplementary Figure S2**). Therefore, a higher risk score is associated with more malignant HCC.

**Figure 4:**
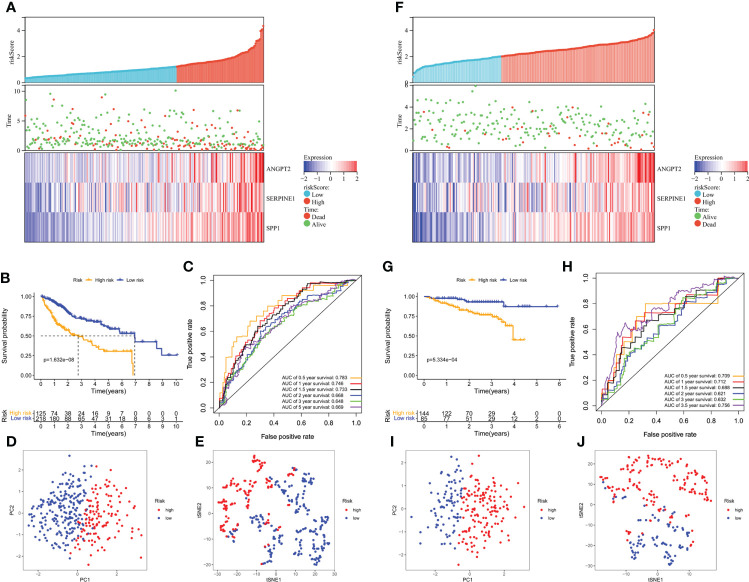
Survival analysis of HCC patients in the TCGA training and ICGC validation datasets. **(A, B)** Risk score distribution, survival status, and heatmap of the expression of the three HRAGs in the high- and low-risk groups in the training and validation cohorts. **(B, G)** Kaplan-Meier OS curve. **(C, H)** AUCs of time-dependent ROC curves. PCA **(D, I)** and t-SNE **(E, J)** analysis confirmed the clustering of the three genes comprising the HRAG signature. **(A–E)** TCGA cohort; **(F–J)** ICGC cohort.

Furthermore, the high-risk group membership was significantly associated with higher tumor grade and advanced tumor, node, metastasis (TNM) stage (**Table 1**). Patients with a high risk score had a higher mortality rate, shorter OS, and higher expression of the three model genes (**Figure 4A**). K-M survival curves indicated significantly longer survival time of the low-risk group, including OS (**Figure 4B**) and disease-free survival (DFS) (**Supplementary Figure S3**), compared to the high-risk group. The AUC of the time-dependent ROC curves was 0.783 at 0.5 years, 0.746 at 1 year, 0.733 at 1.5 years, 0.668 at 2 years, 0.648 at 3 years, and 0.669 at 5 years (**Figure 4C**). PCA (**Figure 4D**) and t-SNE (**Figure 4E**) confirmed risk profile differences between the two groups. To test the robustness of the gene signature model constructed based on the TCGA cohort, patients from the ICGC and GSE14520 cohorts were categorized into high- and low-risk groups according to the optimal cut-off value. The results for the ICGC (**Figures 4G–J**) and GSE14520 cohort (**Supplementary Figure S4**) were similar to those of TCGA cohort. Therefore, the prognostic model was predictive of the prognosis and progression of HCC.

**Table 1:** Baseline characteristics of the patients in different risk groups of three cohorts.

Variables	Group	TCGA cohort (n = 343)		P value	ICGC cohort (n = 229)		P value	GSE14520 cohort (n = 220)		P value
		High risk(n=125)	Low risk(n=218)		High risk(n=144)	Low risk(n=85)		High risk(n=108)	Low risk(n=112)	
median Survival time (days)		412	649		720	900		861	1635	
Survival status	Alive	63(50.4%)	161(73.85%)	**<0.0001**	110(76.39%)	79(92.94%)	**0.0011**	51(47.22%)	85(82.52%)	**<0.0001**
	Dead	62(49.6%)	57(26.15%)		34(23.61%)	6(7.06%)		57(52.78%)	18(17.48%)	
Gender	Female	45(36%)	65(29.82%)	0.2794	39(27.08%)	22(25.88%)	0.8782	12(11.11%)	18(16.07%)	0.3288
	Male	80(64%)	153(70.18%)		105(72.92%)	63(74.12%)		96(88.89%)	94(83.93%)	
Age	≤60	52(41.60%)	113(51.83%)	0.0732	30(20.83%)	19(22.35%)	0.8678	91(84.26%)	90(80.36%)	0.4839
	>60	73(58.40%)	105(48.17%)		114(79.17%)	66(77.65%)		17(15.74%)	22(19.64%)	
Grade	G1	7(5.6%)	46(21.1%)	**0.0009**	/	/		/	/	
	G2	67(53.6%)	94(43.1%)		/	/		/	/	
	G3	48(38.4%)	64(29.4%)		/	/			/	
	G4	2(1.6%)	10(4.6%)		/	/		/	/	
	unknown	1(0.8%)	4(1.8%)		/	/		/	/	
TNMstage	I	43(34.4%)	118(54.2%)	**0.0023**	17(11.8%)	19(22.4%)	**0.0012**	31(28.7%)	62(55.4%)	**<0.0001**
	II	32(25.6%)	45(21.0%)		58(40.3%)	47(55.3%)		39(36.1%)	38(33.9%)	
	III	42(33.6%)	38(17.8%)		53(36.8%)	16(18.8%)		37(34.3%)	11(9.8%)	
	IV	1(0.8%)	2(0.1%)		16(11.1%)	3(3.5%)		0	0	
	unknown	7(5.6%)	15(6.9%)		/	/		1(0.9%)	1(0.9%)	

### Prognostic value of the gene signature

**Figure 5:**
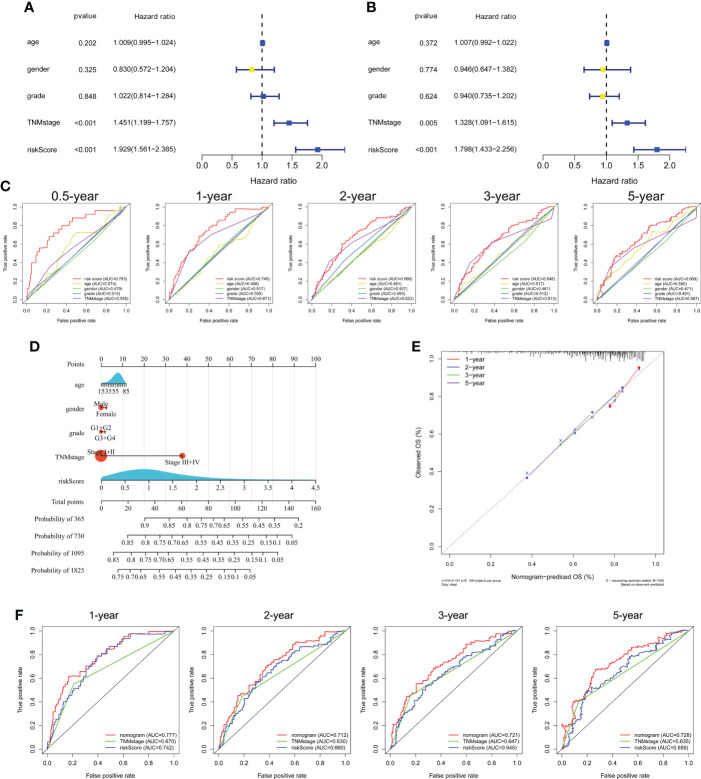
Independent prognostic power of the three genes comprising the signature in the TCGA cohort. **(A)** Univariate and **(B)** multivariate Cox regression analyses of the associations of the risk index (RI) and clinical parameters with OS. **(C)** ROC curve of the prognostic utility of the risk score, age, gender, grade, and TNM stage. **(D)** Nomogram for predicting 1-, 2-, 3-, and 5-year survival. **(E)** Calibration curves of the nomogram showed consistency in the predicted and observed 1-, 2-, 3-, and 5-year survival rates. **(F)** ROC curve analysis of the nomogram for 1-, 2-, 3-, and 5-year OS.

Univariate and multivariate Cox regression analyses showed that TNM stage and risk score were significantly associated with the prognosis of HCC in the TCGA cohort (**Figures 5A, B**). ROC curve analysis showed that the risk score was better for predicting prognosis than the other clinicopathological factors (**Figure 5C**). The risk scores and clinicopathological factors of 343 HCC patients with complete clinical information were used to create a prognostic nomogram for predicting survival (**Figure 5D**). The calibration curves of the prognostic nomogram showed good consistency between the predicted and actual 1-, 2-, 3-, and 5-year survival rates in the TCGA cohort (**Figure 5E**). The nomogram AUCs of the predicted 1-, 2-, 3-, and 5-year OS were 0.777, 0.712, 0.721, and 0.728, respectively (**Figure 5F**).

### Molecular characteristics and biological function analysis

**Figure 6:**
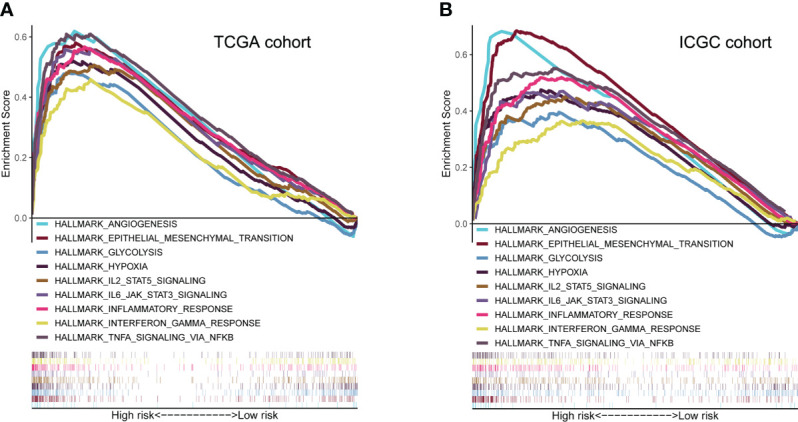
Molecular characteristics and biological function analysis. **(A, B)** Gene set enrichment analysis of biological functions and pathways in the risk score groups (P< 0.05, FDR< 0.25).

GSEA analysis revealed that high-risk group membership was significantly associated with angiogenesis, EMT, glycolysis and hypoxia. The immune-related pathways IL2/STAT5 and IL6/JAK/STAT3, as well as the inflammatory response, interferon (IFN)-γ/response and tumor necrosis factor (TNF)-α signaling *via* NFKB were significantly enriched in the high-risk group (*P*< 0.05, FDR< 0.25) (**Figure 6**).

### Tumor immune microenvironment analysis

**Figure 7:**
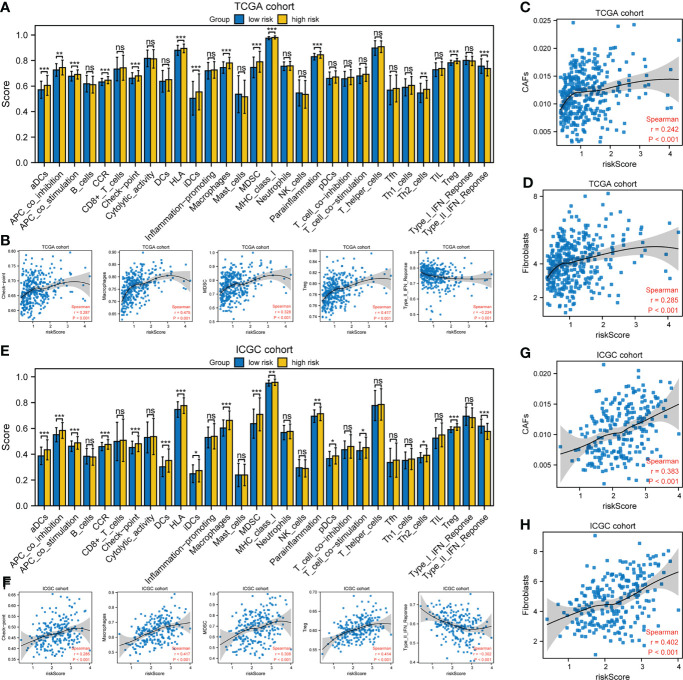
Tumor immune microenvironment analysis. **(A, E)** ssGSEA scores of 17 immune cells and 13 immune-related functions. ns: not significant; *P< 0.05; **P< 0.01; ***P< 0.001. **(B, F)** Spearman’s rank correlation analysis between risk score and immune checkpoints, macrophages, MDSCs, Tregs, and type II IFN response. **(C, D, G, H)** Spearman’s rank correlation analysis between risk score and CAFs according to the **(C, G)** EPIC and **(D, H)** MCPcounter algorithms. **(A–D)** TCGA cohort; **(E–H)** ICGC cohort.

xCell was used to evaluate the proportions of infiltrating immune cells. Patients in the high-risk group had higher proportions of macrophages and T regulatory cells (Tregs) than those in the low-risk group, but there was no difference in the proportions of other immune cell types (**Supplementary Figures S5A, B**). Next, the enrichment scores of immune cell subpopulations, related functions, and pathways were calculated by ssGSEA (**Figure 7**). Interestingly, dendritic cells (DCs), antigen-presenting cells (APCs), human leukocyte antigen (HLA), and major histocompatibility complex class I (MHC class I), which are involved in antigen presentation, were significantly elevated in the high-risk group. Furthermore, the activities of cytokine-cytokine receptors and immune checkpoints, and the enrichment scores for macrophages, myeloid-derived suppressor cells (MDSCs), and Tregs in the high-risk group were higher than in the low-risk group, whereas the type II IFN response showed the opposite trend. The risk score was significantly positively correlated with the proportion of cancer-associated fibroblasts (CAFs). The expression of immunosuppressive genes in the high-risk group was higher than in the low-risk group, in both the TCGA and ICGC cohorts (**Supplementary Figures S5C, E**). The expression of the pro-angiogenic factors, VEGFA and VEGFB, was significantly upregulated in the high-risk group (**Figure S5D, F**). However, there was no difference in the tumor mutation burden (TMB) or microsatellite instability (MSI) score (**Supplementary Figures S5G, H**). Therefore, the TME of patients in the high-risk group is in an immunosuppressive state.

### Prediction and validation of the anti-angiogenesis and ICB responses

**Figure 8:**
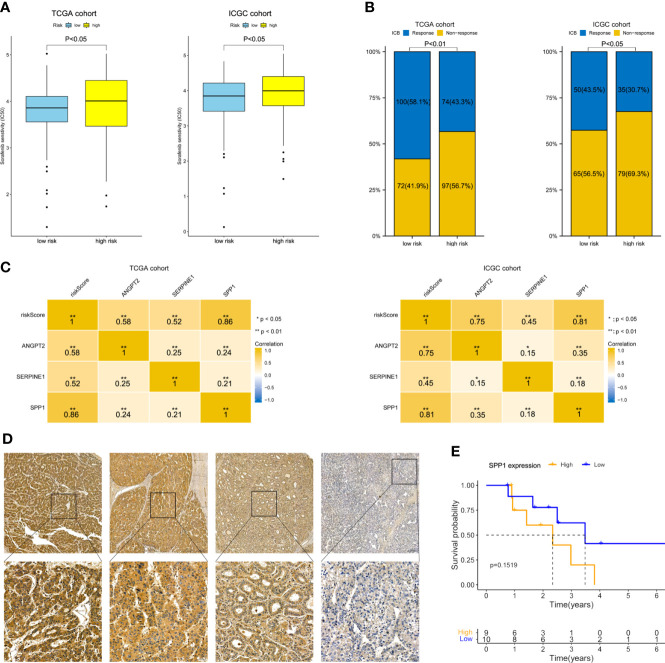
Therapeutic response prediction and immunohistochemistry of SPP1. **(A)** Sensitivity to sorafenib by risk group. **(B)** Response rate to ICB therapy. **(C)** Heatmap showing the correlation between risk score and the three model genes. **(D)** Representative immunohistochemically stained images of HCC tissues showing SPP1 expression (upper, 5.0×; lower, 20.0×). **(E)** K-M OS curves of 19 HCC patients with different SPP1 levels.

As shown in **Figure 8A**, the estimated IC_50_ showed that the low-risk group in both cohorts had a better response to sorafenib (*P<* 0.05). ImmuCellAI showed that the response rate to ICB therapy was higher in the group with a lower risk score (**Figure 8B**). The correlation heatmap indicated that SPP1 expression was most relevant to the risk score, and thus has great potential for predicting the response to anti-angiogenesis and ICB therapy (**Figure 8C**). Next, 19 Barcelona Clinic Liver Cancer (BCLC) C stage-HCC samples subjected to anti-angiogenesis treatment and immunotherapy after resection were further subjected to IHC staining. Follow-up information was collected from January 2015 to April 2022. Based on the H-scores, we divided the 19 patients into high- and low-expression groups. Representative immunohistochemically stained images are shown in **Figure 8D**. K-M curves showed that the OS of the low-expression group was trend longer than that of the high-expression group though not statistically significant (P = 0.1519, **Figure 8E**). In summary, the gene signature was predictive of the response to anti-angiogenesis treatment and ICB therapy.

## Discussion

Predicting the effect of HCC treatment is challenging due to the paucity of useful biomarkers. The American Joint Committee on Cancer (AJCC) TNM staging system has long been considered reliable for predicting the prognosis of patients with HCC. However, it is based on macroscopic information, which does not reflect the biological features and heterogeneity of HCC. Integration of prognostic gene signatures and traditional parameters has advantages for predicting the prognosis of HCC. The importance of predicting the prognosis of HCC and administering treatments in a timely manner highlights the need to identify robust prognostic biomarkers for risk stratification.

Few studies have focused on the predictive value of hypoxia- or angiogenesis-related signatures. The complex regulatory mechanisms and effects of hypoxia-related angiogenesis genes (HRAGs) give rise to an ambiguous relationship with the prognosis of HCC. To our knowledge, this study is the first to develop a novel scoring system based on HRAGs. We investigated the expression of HRAGs in HCC tumor tissues, and their associations with survival, and constructed a novel prognostic model based on three HRAGs (ANGPT2, SERPINE1, and SPP1) to stratify HCC patients according to their estimated survival. The prognostic model showed good predictive power in both the training cohort and two external validation cohorts. Univariate and multivariate Cox regression analyses revealed the independent prognostic value of the three genes comprising the gene signature. A nomogram showed that the gene signature was predictive of 1-, 2-, 3-, and 5-year OS, and so may be useful when planning short-term follow-up. However, only three databases were used in this study, and the signature needs to be validated in an independent cohort.

The prognostic gene signature comprised three HRAGs (ANGPT2, SERPINE1, and SPP1). ANGPT2, which belongs to the angiopoietin family, is highly expressed in diverse tumor cells, and is implicated in tumor angiogenesis and inflammation (20, 21). ANGPT2 is highly expressed in, and closely related to, the development and prognosis of, HCC (15, 22). Increased expression of plasminogen activator inhibitor type 1 (also known as SERPINE1) was associated with tumor cell migration and invasion *via* activation of the PI3K-Akt pathway (23, 24). High expression of SERPINE1 in cancer tissues predicts a poor clinical outcome. In this study, SERPINE1 expression was lower in HCC tumor tissues than adjacent normal tissues in the TCGA dataset (**Figure 2**), possibly because of the different roles SERPINE1 in tumor and normal tissues. SPP1, also called osteopontin, is an arginine-glycine-aspartate-containing phosphoprotein that is overexpressed in many cancers, including lung adenocarcinoma and HCC, and serves as a prognostic biomarker (25–27). SPP1 is involved in tumor immunosuppression and affects the TME (28). The expression of the three prognostic model genes, and the risk score, increased with increasing tumor stage, suggesting that they correlate with tumor malignancy and progression (**Supplementary Figure S2**).

To identify the pathway involved in hypoxia-related angiogenesis, HCC patients were divided into two groups according to the median risk score calculated by GSEA. Biological function analysis revealed greater activity not only in hypoxia- and angiogenesis-related pathways, but also in the EMT pathway (**Figure 6**). The EMT is a process of phenotypic plasticity that has roles in organ development, wound healing, tumor progression, and the response to therapeutics (29). Our findings suggest that HRAGs promote HCC progression by regulating the EMT pathway.

Because of its importance for immunotherapy and, potentially, precision therapy, the TME is a focus of research (30). In terms of predictive biomarkers, our high-risk group had higher proportions of macrophages, Tregs, and MDSCs (**Figure 7**). Higher proportions of tumor-associated macrophages and Tregs are associated with a poor prognosis of HCC (31). In addition, impairment of the type II IFN response and increased infiltration of MDSCs, Tregs, and macrophages, as in the high-risk group, is implicated in tumor immunological escape and tolerance, and impairs the antitumor T-cell response in HCC (32–34). Furthermore, the risk score was positively related to the proportion of CAFs in HCC. As a critical component of the TME, CAFs contribute to immune evasion and immunotherapy failure, and promote the proliferation and invasion of tumors, including HCC, by secreting growth factors and cytokines (35–37). The immune-related pathways IL2/STAT5 and IL6/JAK/STAT3, as well as the inflammatory response, IFN-γ response, and TNFα signaling *via* NFκB, were significantly enriched in the high-risk group (**Figure 6**). Cancer cells may drive the expression of immune checkpoints *via* these immune-related pathways (38). The expression of immune-related checkpoints (PD-L1, LAG3, CTLA4, TIM3, and TIGIT) in our high-risk group was higher than in the low-risk group, but the MSI and TMB, which indirectly reflect the ability of a tumor to produce new antigens and predict the efficacy of immunotherapy for a variety of tumors, were not significantly different between the groups (**Supplementary Figure S5**). The expression of the pro-angiogenic factors VEGFA and VEGFB was significantly upregulated in the high-risk group in the ICGC and TCGA databases; these cytokines promote TME remodeling and directly or indirectly regulate immune cells (17). Therefore, in the high-risk group, anti-tumor immunity is probably attenuated; also, a high risk score may be correlated with immunosuppression in HCC, possibly explaining its poor prognosis. This may arise because the expression of angiogenesis-related genes under hypoxic conditions induces the production of myeloid suppressor cells, Tregs, and immunosuppression-related checkpoints, thereby disrupting the immune balance. In summary, tumor-driven hypoxia related angiogenesis plays a crucial role in modulating the tumor immune microenvironment.

In view of the fact that angiogenesis plays a central role in immunosuppression and can lead to resistance to ICBs, there is growing evidence to support a strategy combining anti-angiogenesis and ICBs with promising clinical activity. Due to the encouraging efficacy and safety findings of the IMbrave150 trial for atezolizumab plus bevacizumab, this novel anti-angiogenesis combined with immunotherapy has become the first-line treatment for patients with unresectable HCC (39, 40). The combination therapy can render the TME conducive to immune cell function. However, because angiogenesis and the TME have multiple roles, the mechanism underlying the effect of anti-angiogenesis combined with immunotherapy on liver cancer is unclear.

Our findings suggest that HRAGs not only contribute to tumor growth, metastasis, progression, and poor prognosis, but also to the infiltration of immunosuppressive cells and high expression of immune checkpoints. This could explain why anti-angiogenesis combined with immunotherapy is effective for unresectable HCC. The anti-angiogenesis and ICB therapy response rate was higher in the low-risk group. And SPP1 expression was most relevant to the risk score. Previous studies have illustrated that tumor-driven hypoxia promotes the expression of SPP1, which in turn promotes tumor angiogenesis and immunosuppressive microenvironment (28, 41, 42). SPP1 may be considered as a general marker of cancer progression, would be valuable in combination with other biomarkers to guide patient stratification and treatment strategies, and would be an attractive therapeutic target due to its multiple roles in promoting tumor aggressiveness. Our present study interestingly found that the OS of the low-SPP1-expression group of HCC patients who received anti-angiogenesis combined with immunotherapy after resection was trend longer than that of the high-SPP1-expression group (**Figure 8**). These findings may aid the development of comprehensive therapeutic strategies for HCC.

This study also had some limitations. First, the gene signature model was constructed and validated based on retrospective data from public databases. Second, the small sample size hampered statistical analysis. Third, a further study should investigate the biological mechanisms underlying the signature.

## Conclusion

A novel gene signature model and HRAGs-based prognostic biomarker were developed and validated in an independent cohort. The gene signature may help identify immune infiltration and predict sensitivity to anti-angiogenesis and immunotherapy, and the outcomes of HCC. The mechanism underlying the associations of HRAGs expression in HCC with the TME and sensitivity of anti-angiogenesis and immunotherapy are unclear, so further studies are needed.

## Data availability statement

The datasets presented in this study can be found in online repositories. The names of the repository/repositories and accession number(s) can be found below: TCGA-LIHC: https://portal.gdc.cancer.gov/; ICGC: https://dcc.icgc.org/projects/LIRI-JP; GEO: https://www.ncbi.nlm.nih.gov/geo/query/acc.cgi?acc=GSE14520.

## Ethics statement

The studies involving human participants were reviewed and approved by the Institutional Review Boards of the Research Institute and Hospital National Cancer Center and The First Affiliated Hospital, Sun Yat-Sen University. The patients/participants provided their written informed consent to participate in this study.

## Author contributions

JL, GZ and YX conceived and designed the project. JL supervised the study. GZ and YX conducted and performed data collection and data analysis and interpretation. XZ, WF, YZ, YW, and HW contributed to discussion and reviewed and edited the manuscript. All authors read and approved the final version of the manuscript.

## Funding

This research was supported by the National Natural Science Foundation of China (NSFC) (82172036, 81971719 and 81171441), and the Key Research and Development Project of Guangzhou City (202103000021), and the major scientific and technological project of Guangdong Province (2020B0101130016), and the National Natural Science Foundation of China (Youth Project, 82102161).

## Conflict of interest

The authors declare that the research was conducted in the absence of any commercial or financial relationships that could be construed as a potential conflict of interest.

## Publisher’s note

All claims expressed in this article are solely those of the authors and do not necessarily represent those of their affiliated organizations, or those of the publisher, the editors and the reviewers. Any product that may be evaluated in this article, or claim that may be made by its manufacturer, is not guaranteed or endorsed by the publisher.

## Abbreviations

AUC, Areas under the curve; CAFs, Cancer-associated fibroblasts; DEGs, Differentially expressed genes; DFS, disease free survival; FDG, false discovery rate; GEO, Gene-Expression Omnibus; GSEA, Gene Set Enrichment Analysis; HCC, hepatocellular carcinoma; HRAGs, Hypoxia-related angiogenesis genes; ICB, Immunological checkpoint blockade; ICGC, International Cancer Genome Consortium; MDSCs, Myeloid-derived suppressor cells; OS, overall survival; PCA, Principal component analysis; ROC, Receiver operator characteristic; ssGSEA, Single-sample gene-set enrichment analysis; TCGA, The Cancer Genome Atlas; TME, Tumor microenvironment; t-SNE, t-distributed stochastic neighborembedding.
