# Supplementary material for: Dissecting a hypoxia-related angiogenic gene signature for predicting prognosis and immune status in hepatocellular carcinoma
Source: Front Oncol. 2022 Aug 30;12:978050. doi: 10.3389/fonc.2022.978050 (PMC9468769; doi:10.3389/fonc.2022.978050)
Supplement: Supplementary file 1 [file DataSheet_1.docx]

**Dissecting a Hypoxia-related Angiogenic Gene Signature for Predicting Prognosis and Immune status in Hepatocellular Carcinoma**

**Supplementary Figures**


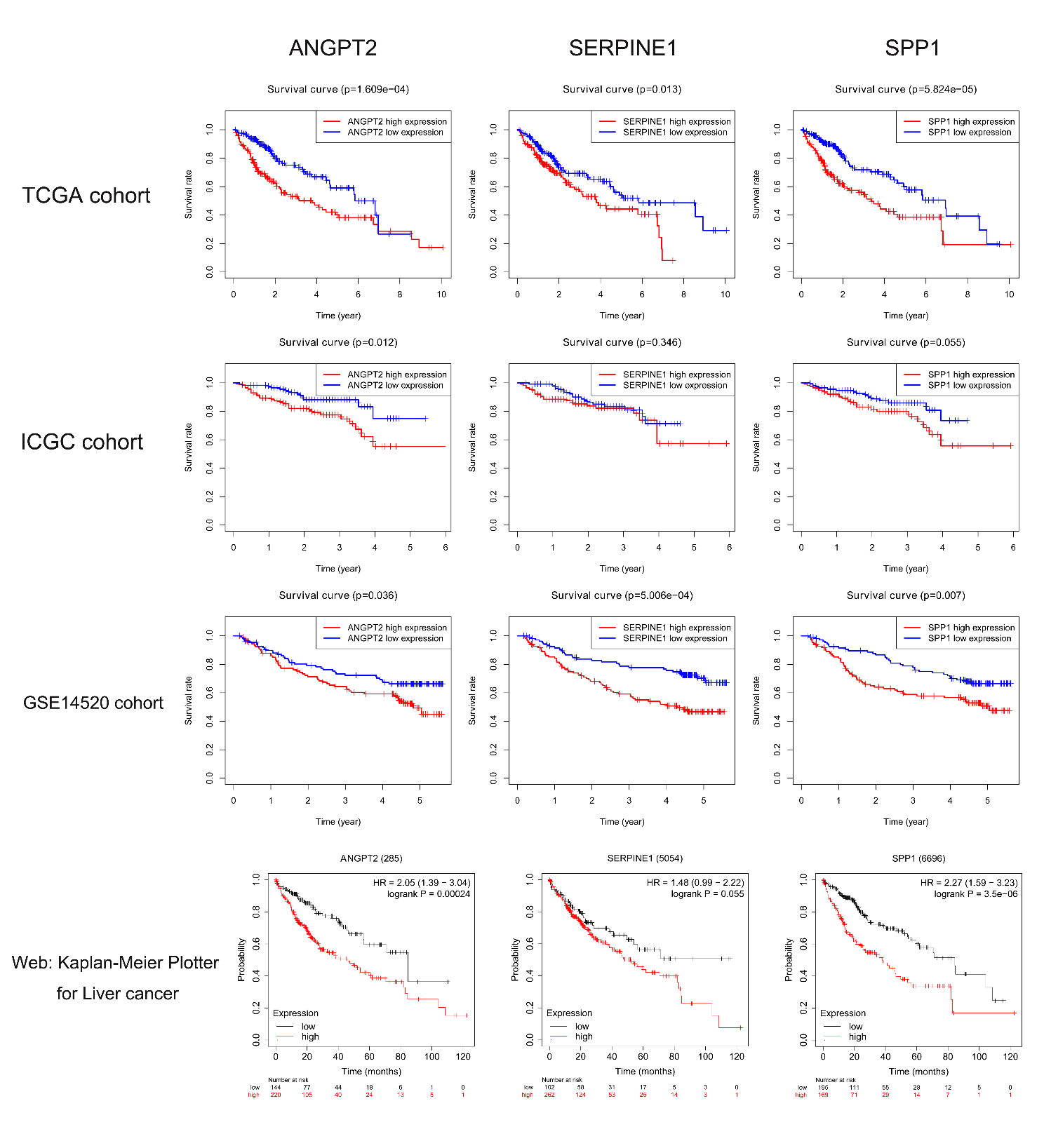


**Figure S1. Kaplan-Meier OS curves based on the three model genes.**


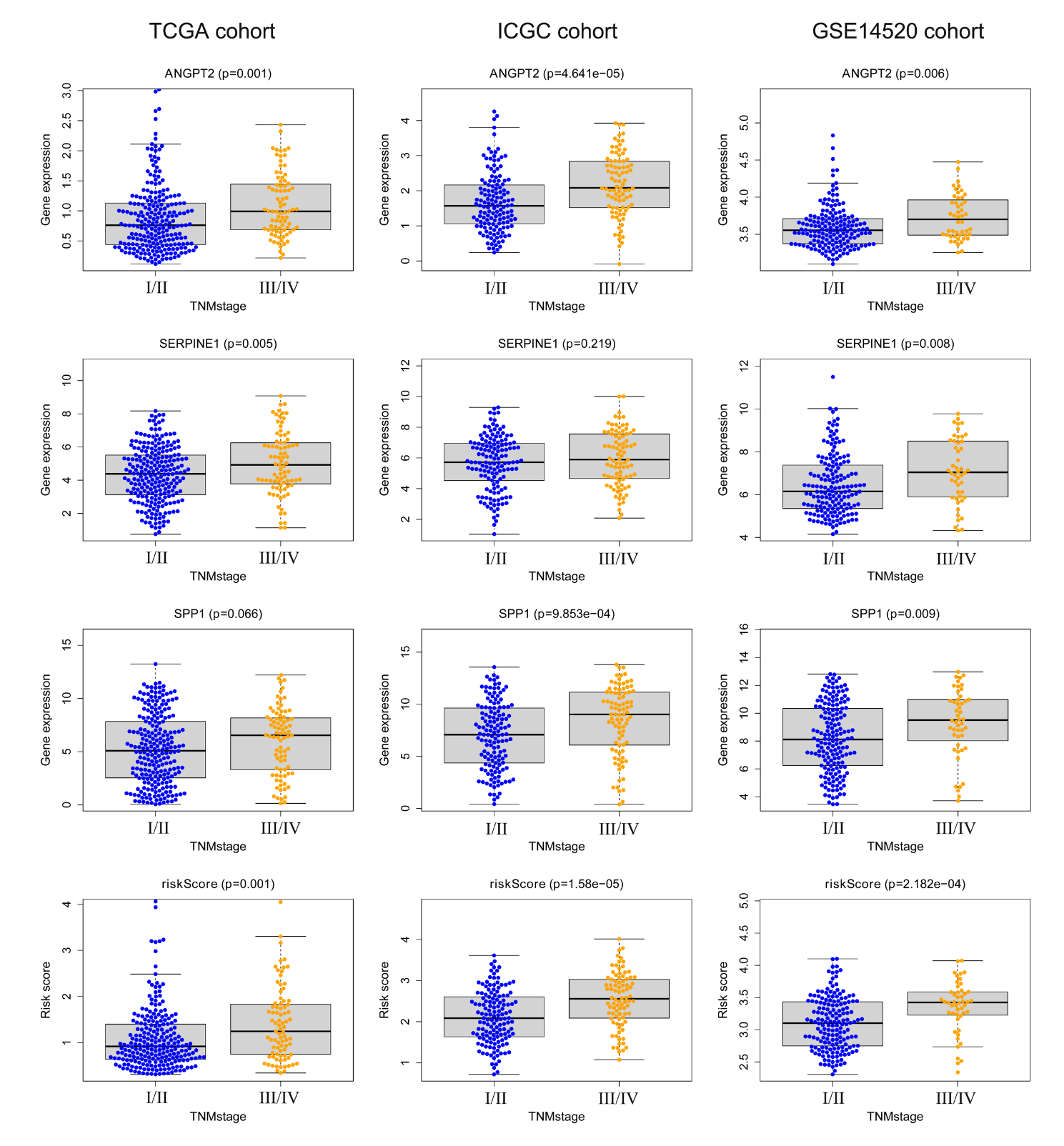


**Figure S2. ANGPT2, SERPINE1, and SPP1 gene expression and risk score by tumor stage.**


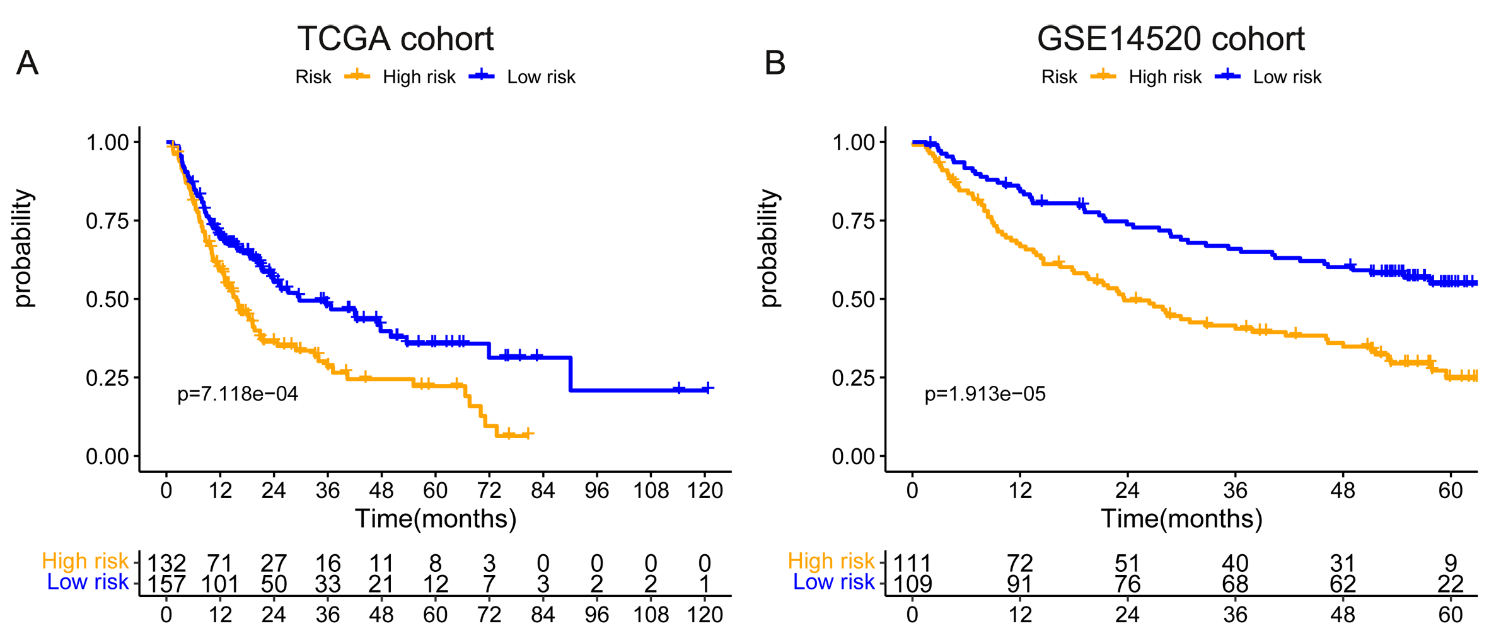


**Figure S3. Kaplan-Meier DFS curves for the high- and low-risk groups.**


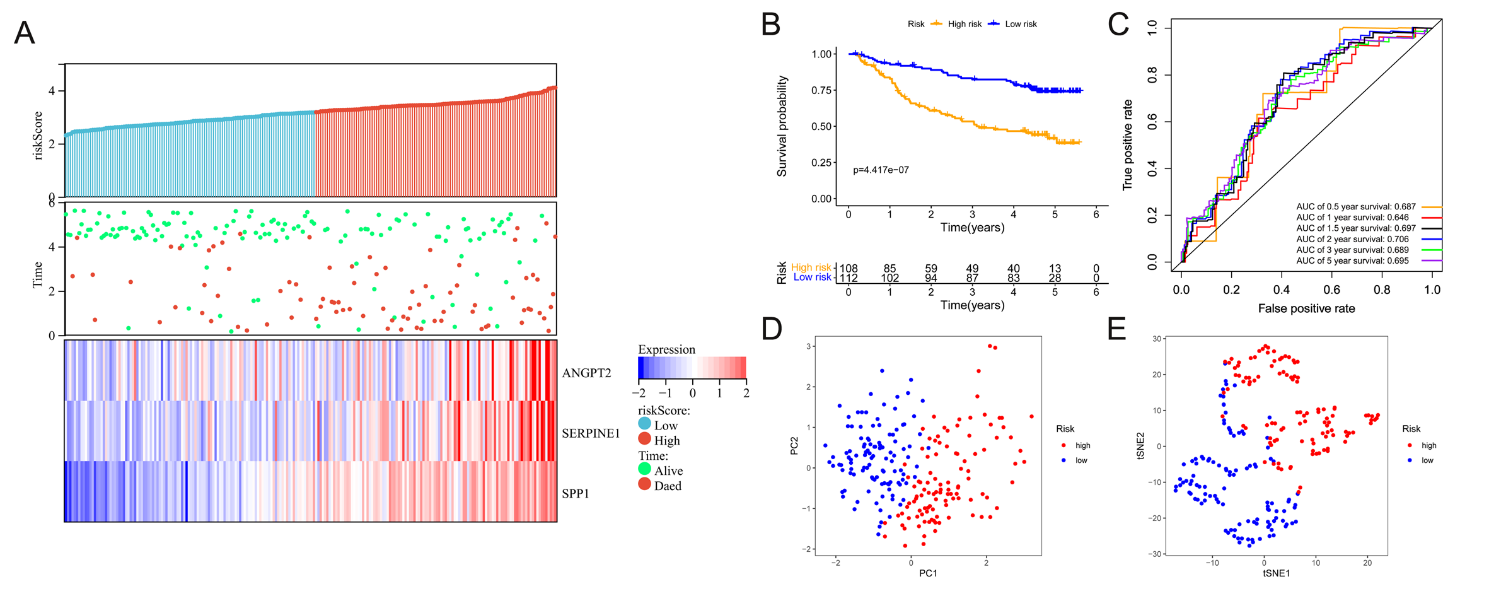


**Figure S4.** **Survival of HCC patients in the GSE14520 validation dataset.** (A) Risk score distribution, survival status, and heatmap of the expression of the three HRAGs in the high- and low-risk groups. (B) Kaplan-Meier curves for OS. (C) AUC of time-dependent ROC curves. PCA (D) and t-SNE (E) analysis confirmed the clustering of the three genes comprising the HRAG signature.


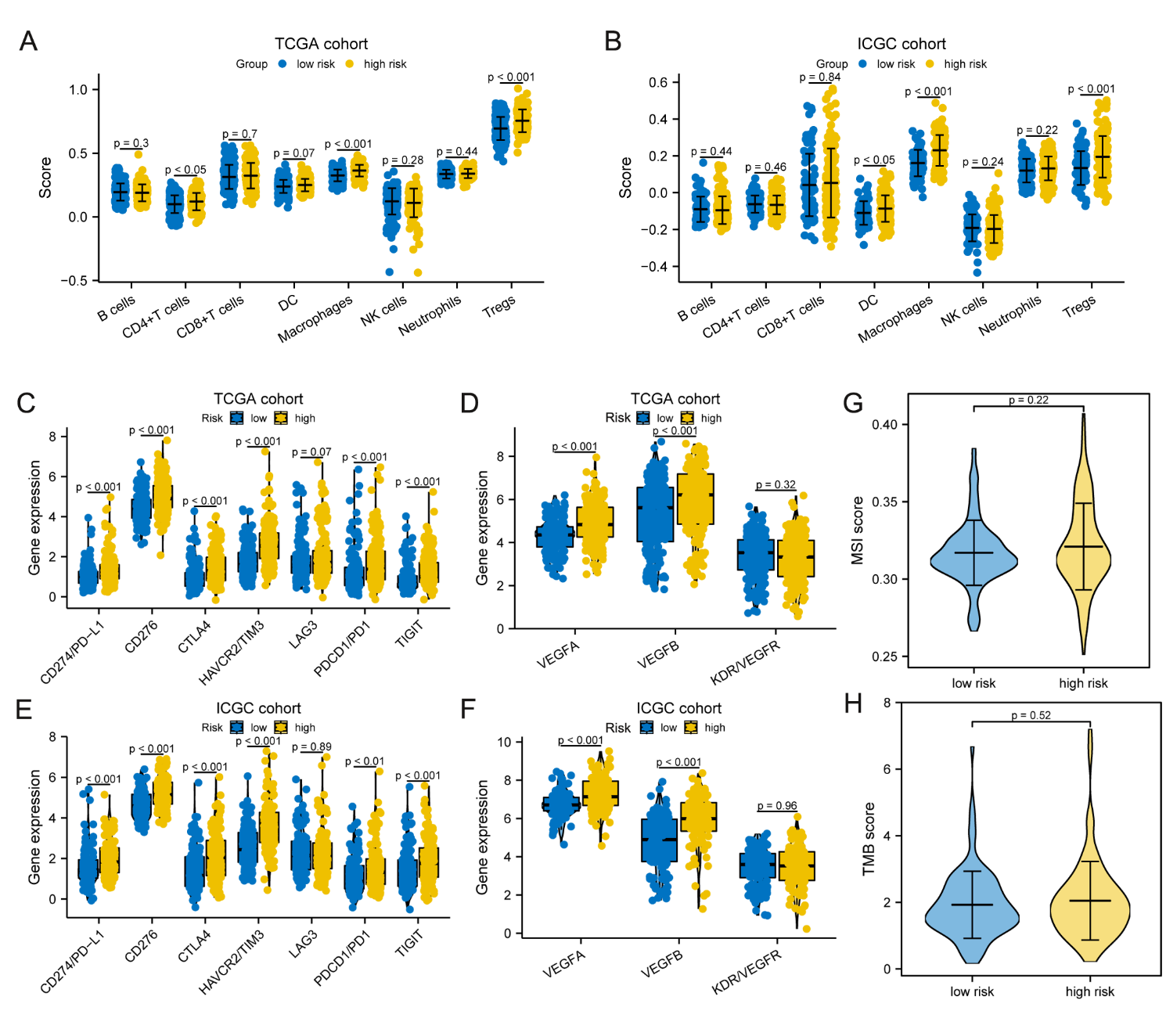


**Figure S5. Tumor immune microenvironment.** (A, B) Infiltrating immune cell analysis by xCell. (C, E) CTLA-4, LAG-3, PD-1, TIGIT, TIM-3, CD276, and PD-L1 expression. (D, F) VEGFA and VEGFB expression. (G, H) TMB and MSI score.


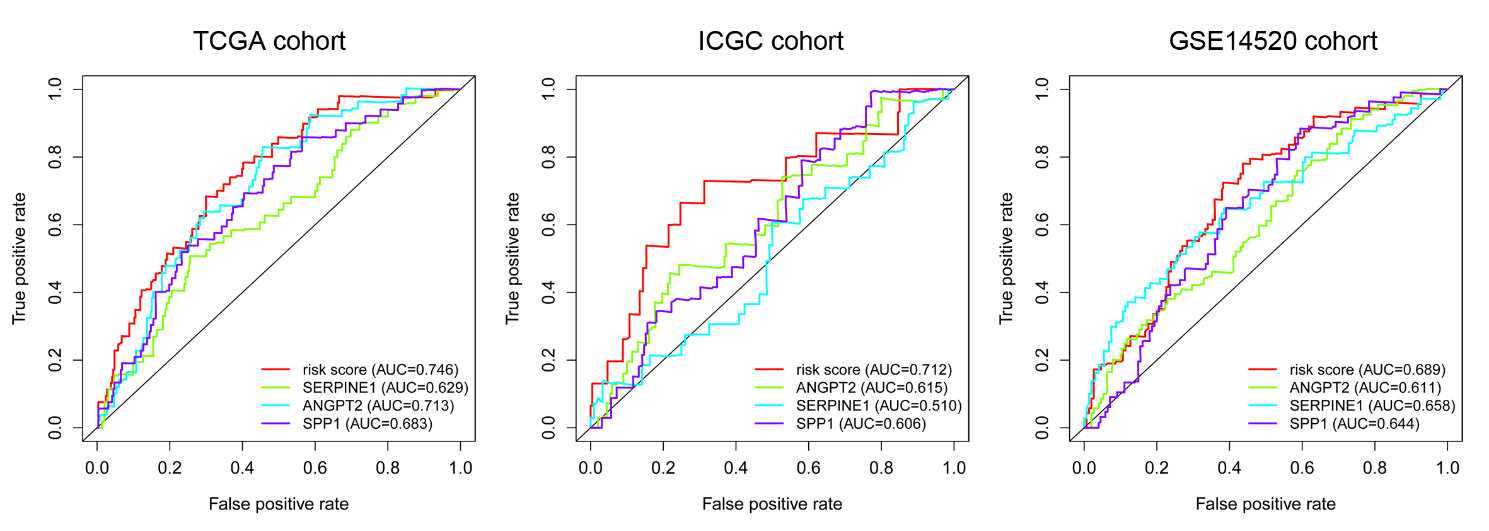


**Figure S6.** **AUC of ROC curves indicated that the prognostic predictability of the gene signature built with three genes was better than a single gene.**

Table S1. Patient demographics and clinical characteristics of the included datasets.

| Variables | Group | TCGA cohort (n = 343) | ICGC cohort (n = 229) | GSE14520 cohort (n = 220) |
| --- | --- | --- | --- | --- |
|  |  |  |  |  |
| median Survival time (days) |  | 587 | 780 | 1570 |
|  |  |  |  |  |
| Survival status | Alive | 224 (65%) | 189 (83%) | 136 (62%) |
|  | Dead | 119 (35%) | 40 (17%) | 84 (38%) |
| Gender | Female | 110 (32%) | 61 (27%) | 30 (14%) |
|  | Male | 233 (68%) | 168 (73%) | 190 (86%) |
| Age | ≤60 | 165 (48%) | 49 (21%) | 181 (82%) |
|  | >60 | 178 (52%) | 180 (79%) | 39 (18%) |
| Tumor grade | G1 | 53 (15%) | / |  |
|  | G2 | 161 (47%) | / |  |
|  | G3 | 112 (33%) | / |  |
|  | G4 | 12 (3.5%) | / |  |
|  | Unknown | 5 (1.5%) | / |  |
| TNM staging | I | 161 (47%) | 36 (16%) | 93 (42%) |
|  | II | 77 (23%) | 105 (46%) | 77 (35%) |
|  | III | 80 (23%) | 69 (30%) | 48 (22%) |
|  | IV | 3 (1%) | 19 (8%) | / |
|  | Unknown | 22 (6%) | / | 2 (1%) |
